# Seizures and risks for recurrence in critically ill patients: an observational cohort study

**DOI:** 10.1007/s00415-022-11038-6

**Published:** 2022-03-02

**Authors:** Anna S. Wagner, Saskia Semmlack, Anja Frei, Stephan Rüegg, Stephan Marsch, Raoul Sutter

**Affiliations:** 1 Department of Neurology, University Hospital Basel, Basel, Switzerland; 2 Department of Anesthesiology, University Hospital Basel, Basel, Switzerland; 3 Department of Intensive Care, University Hospital Basel, 4031 Basel, Switzerland; 4 Medical Faculty, University of Basel, Basel, Switzerland

**Keywords:** Seizures, Seizure recurrence, Acidosis, Intensive care units, Neurocritical care

## Abstract

**Background:**

To assess the frequency and clinical characteristics of seizures in adult critically ill patients, to identify predictors of recurrent seizures not transforming into status epilepticus and to characterize their effects on course and outcome.

**Methods:**

ICU patients at a Swiss academic medical center with seizures not transforming into status epilepticus from 2015 to 2020 were included. Recurrent seizures and associated clinical characteristics were primary, death, and return to premorbid neurologic function were secondary outcomes.

**Results:**

Two hundred of 26,370 patients (0.8%) with a median age of 65 years had seizures during ICU stay. Seizure semiology was described in 82% (49% generalized; 33% focal) with impaired consciousness during seizures in 80% and motor symptoms in 62%. Recurrent seizures were reported in 71% (36% on EEG) and associated with longer mechanical ventilation (*p* = 0.031), higher consultation rate by neurologists (*p* < 0.001), and increased use of EEG (*p* < 0.001) when compared to single seizures. The use of EEG was not associated with secondary outcomes. Acidosis at seizure onset and prior emergency operations were associated with decreased odds for seizure recurrence (OR 0.43; 95% CI 0.20–0.94 and OR 0.48; 95% CI 0.24–0.97). Epilepsy had increased odds for seizure recurrence (OR 3.56; 95% CI 1.14–11.16).

**Conclusions:**

Seizures in ICU patients are infrequent, but mostly recurrent, and associated with higher resource utilization. Whenever seizures are observed, clinicians should be vigilant about the increased risk of seizures recurrence and the need for antiseizure treatment must be carefully discussed. While known epilepsy seems to promote recurrent seizures, our results suggest that both acidosis and previous emergency surgery seem to have protective/antiseizure effects.

**Trial registration:**

Clinicaltrials.gov (No. NCT03860467).

## Background

While the frequency and systemic and cerebral impact of seizures that transform into status epilepticus in critically ill patients is well known [19], little is known about the effects of single and recurrent seizures without transformation into status epilepticus. There are only few studies on seizures in critically ill patients treated in intensive care units (ICUs) reporting 1–40% of critically ill patients having seizures depending on the awareness of the treating teams, the investigated cohorts, and the electrophysiologic monitoring [18, 21]. Limited evidence suggests no clear associations between seizures and specific outcomes [17], but large studies regarding the promotors of single or recurrent seizures and their impact on the course of underlying diseases, resource utilization, and management of critically ill patients are lacking.

We aimed to assess the frequency and clinical characteristics of seizures in adult critically ill patients, to identify predictors of recurrent seizures not transforming into status epilepticus and to characterize their effects on course and outcome during intensive medical care.

## Material and methods

This study was performed at the University Hospital of Basel, a Swiss tertiary academic medical center and registered prior to data assessment (ClinicalTrials.gov NCT03860467). We followed the STROBE (STrengthening the Reporting of OBservational studies in Epidemiology)-guidelines to enhance the quality of our study [25]. The local ethics committee approved the study (Ethikkommission Nordwestschweiz [EKNZ] approval number 2018-02361) and patients’ consent was waived.

### Data collection

Medical and electroencephalographic (EEG) records of all adult (≥ 18 years of age) ICU patients from January 1st 2015 to December 31st 2020 with reported seizures not transforming into status epilepticus were retrospectively assessed. The following data were collected from the prospectively recorded digital EEG database and the ICU information system MetaVision (iMDsoft, Wakefield, MA) and entered into a predefined case report form: demographics, presumed seizure etiology (categorized as potential nonfatal and fatal as defined in recent studies on status epilepticus [15]), the Charlson Comorbidity Index [2], the Acute Physiology and Chronic Health Evaluation-II (APACHE II) score [9], the Sequential Organ Failure Assessment (SOFA) score [24], reported seizure semiology, such as focal or bilateral seizures, seizures with impaired consciousness, and with or without motor symptoms. To comply with the American Clinical Neurophysiology Society (ACNS) guidelines, the term bilateral seizures was used instead of “generalized” seizures, as the latter is reserved for primary generalized seizures. Treatment characteristics included duration of mechanical ventilation, the use of antiseizure drugs prior and in response to seizure, administered anesthetics and vasopressors, consultation by neurologists, duration of hospital and ICU stay. Complications in close temporal relation to seizures were noted, such as infections diagnosed within 7 days prior seizure onset, blood gas analyzes at seizure onset, and persistent postictal coma for at least 24 h. A protocol for monitoring infections was followed as described in prior studies [1, 16, 22] according to the CDC [3]. We assessed the performance of spot EEGs for 30–60 min 24 h around seizures or immediately (within several minutes) after seizures, and the use of continuous EEG (cEEG) for > 1 h, as well as EEG features categorized as signs of encephalopathy, focal slowing, and epileptic seizures according to the ACNS [6]. As routine clinical practice, all patients with clinical, electrographic or electro-clinical seizures and no complete recovery of neurologic function and consciousness within 30 min following seizure received EEGs. Continuous EEG was initiated if initial spot EEG was abnormal with epileptic potentials not qualifying as epileptic seizures and/or the patients remained in a state of not otherwise explained impaired consciousness or altered neurologic function during and/or after the first EEG. All EEGs were analyzed by two board certified EEGers and in cases of disagreement consensus was reached by joint review.

Outcome at hospital discharge, such as return or no return to premorbid neurologic function and death were assessed.

### Single and recurrent seizures

Patients were categorized as having seizures, based on one or more of the following criteria: (1) a clear and detailed clinical description of motor symptoms typically seen with epileptic seizures (according to the International League Against Epilepsy [ILAE]; outlined below) by the treating neurologists and/or intensivists including postictal cessation of symptoms and improvement of consciousness and/or neurologic function during ICU stay; (2) EEG immediately following the first seizure had to detect repetitive epileptiform discharges (spikes and/or sharp waves); (3) EEG detecting epileptic seizures after clinical full recovery to neurologic baseline following first seizure.

Motor symptoms typically seen with epileptic events were the following according to the ILAE diagnostic manual and involving body parts according to their representation on the motor cortex. Such focal or bilateral symptoms included the following: rhythmic myocloni; Jacksonian march; tonic muscle contractions; sustained contractions of agonist and antagonist muscles producing athetosis or twists; clusters of muscle contractions for milliseconds (jerks); sudden loss/diminution of muscle tone; flexion, extension or mixed flexion–extension for seconds (spasms) of proximal and truncal muscles in series; pedaling, pelvic thrusting, jumping, thrashing and/or rocking movements (hyperkinetic movements); repetitive activity, resembling voluntary movements, but undertaken without volition (automatisms); dysarthria/anarthria while language functions are intact; sustained, forced conjugate ocular, cephalic, and/or truncal rotation or lateral deviation (left or right).

Patients with seizures lasting > 5 min and/or recurrent seizures without full interictal recovery of consciousness or neurologic function to the pre-seizure level over a period of > 5 min were diagnosed with status epilepticus and were excluded. Seizure semiology reports were finally based on the expert analyses of the video-EEG footage (if available) and clinical notes. Semiologic reports were categorized into focal or focal to bilateral, and with and without motor symptoms.

### Outcomes

Primary outcomes were defined as recurrent seizures during ICU stay and associated clinical characteristics. Death and return to premorbid neurologic function were defined as secondary outcomes.

### Statistics

Patients were categorized into patients with and without recurrent seizures during ICU stay. Chi-squared or Fisher’s exact test (for small sample sizes) were used for univariable comparisons of proportions. Continuous variables were analyzed using the Student *t* test for normal distributions and the Mann–Whitney *U* test for non-normal distributions. Discrete variables were expressed as counts, and continuous variables as medians and interquartile ranges. All variables with significant differences between patients with or without recurrent seizures were included in the logistic regression analyses. Multivariable logistic regression analysis was performed to identify variables associated with in- or decreased odds for recurrent seizures during ICU stay. To check the multivariable logistic regression models, Hosmer–Lemeshow *χ*^2^ goodness-of-fit tests were performed. These tests provide summary measures of calibration based upon a comparison of observed and estimated outcomes. To identify possible correlations between clinical parameters and the number of patients with recurrent seizures, linear regression analyses were performed.

Two-sided *p* values ≤ 0.05 were considered significant.

Statistics were performed with STATA^®^16.1 (Stata Corp., College Station, TX, USA).

### Data availability

The corresponding author has full access to the data. He takes responsibility for the integrity of the data, the accuracy of the analysis and interpretation, and the conduct of the research. The authors have the right to publish all data, separate, and apart from the guidance of any sponsor.

## Results

**Fig. 1:**
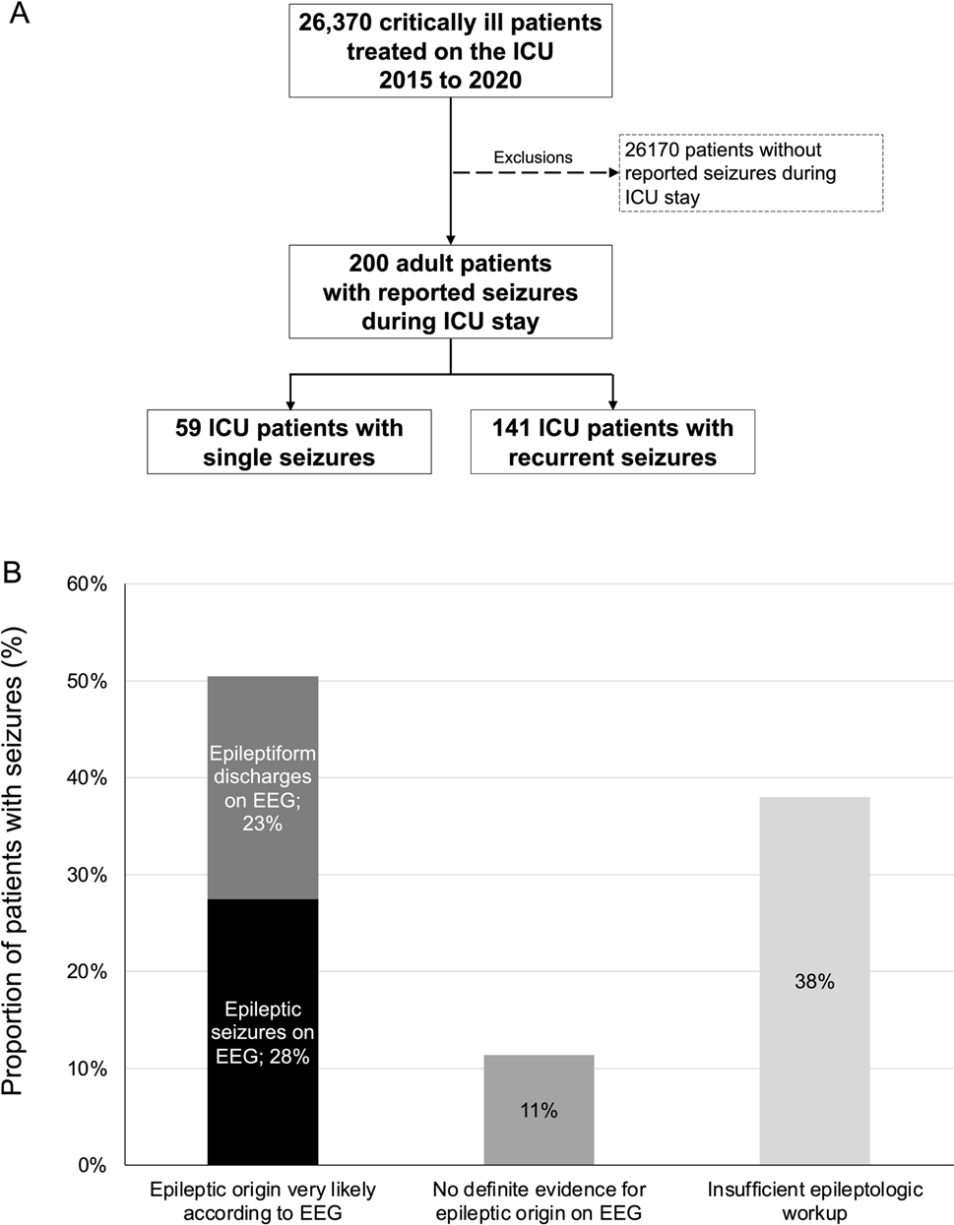
Flow chart (**A**) and evidence from epileptologic workup (**B**). ICU intensive care unit

Among 26,370 patients, seizures were reported in 200 (0.8%) with a median age of 65 years. Details regarding in- and exclusion of patients with seizures during intensive care are presented in Fig. 1A. Demographics, baseline characteristics, monitoring, and treatment measures are outlined in Table 1. Seizure semiology was described in 82% (49% generalized; 33% focal) with impaired consciousness during seizures in 80%, motor symptoms in 62%, and no motor symptoms in 20%. EEGs were performed 24 h around seizures in 76% and immediately following seizures in 62%, with continuous EEG in 28%. EEG was not performed 24 h around seizures in patients in whom the neurologists and intensivists were reporting motor symptoms typically seen with epileptic seizures as described above.

**Table 1:** Baseline characteristics, monitoring, and treatment measures of ICU patients with seizures (*n* = 200)

Demographics and admission characteristics	*n*/median	%/IQR
Age (years; median, IQR)	65	52–75
Female (*n*, %)	88	44
Charlson Comorbidity Index (median, IQR)	5	3–7
APACHE II	24	18–28
SOFA	7	4–9
Presumed seizure etiology (not mutually exclusive; *n*, %)		
Confirmed potential fatal etiology	161	80.5
Acute hemorrhagic or ischemic stroke	87	43.5
Hypoxic ischemic encephalopathy	35	17.5
Encephalitis/meningitis	21	10.5
Septic encephalopathy	19	9.5
Brain tumor	18	9.0
Intoxication	6	3.0
Known epilepsy	43	21.5
Seizure type and characteristics		
Recurrent seizures (*n*, %)	141	70.5
Seizures reported to be focal (*n*, %)	66	33.0
Seizures reported to be focal to bilateral (*n*, %)	98	49.0
Seizures reported with motor symptoms (*n*, %)	123	61.5
Seizures reported without motor symptoms (*n*, %)	39	19.5
Seizure reported with altered consciousness (*n*, %)	160	80.0
Lowest GCS on day of seizure (points; median, IQR)	5	3–10
EEG 24 h around seizure	152	76.0
EEG immediately after or during seizure	124	62.0
Continuous EEG	55	27.5
EEG with generalized slowing	111	55.5
EEG with focal slowing and	59	29.5
EEG with epileptiform discharges (spikes or sharp waves)	46	23.0
EEG with epileptic seizures	55	27.5
Treatment characteristics during seizure		
Seen by neurologist (*n*, %)	165	82.5
In-hospital treatment (days; median, IQR)	15	9–26
ICU treatment (days; median, IQR)	5	2–10
Patients with previous antiseizure drugs (*n*, %)	103	51.5
New or antiseizure drugs given in response to seizure (*n*, %)	144	72.0
Patients with intravenous anesthetic drugs during seizure (*n*, %)	85	42.5
Duration of mechanical ventilation (days; median, IQR)	4	1–8
Outcomes (*n*, %)		
No return to premorbid neurologic function at discharge (incl. death)	153	76.5
Death at hospital discharge	42	21.0

*IQR* interquartile range, *GCS* Glasgow coma score, *EEG* electroencephalography, *APACHE* *II* acute physiology and chronic health evaluation II (range 0–71), *SOFA* sequential organ failure assessment (range 0–24), *ICU* intensive care unit

In 51% of patients with observed seizures (including 36 patients without reported semiology), EEG detected epileptiform discharges or epileptic seizures. In the 24% without EEG 24 h around seizures, semiology was clearly described by the treating neurologists or intensivists including postictal recovery of consciousness and/or neurologic function within the same day. Figure 1B presents the performance and findings of the epileptologic workup with epileptiform discharges or epileptic seizures in 51% of patients and insufficient epileptologic workup in 38% (*n* = 72). Of all patients, 145 (72.5%) received new antiseizure drugs. Recurrent seizures were reported in 141 patients (71% of seizing patients with 39% [55/141 patients] on EEG).

### Univariable comparisons

Table 2 presents the comparisons of baseline characteristics of patients with single and recurrent seizures. Demographics did not differ among patients with and without recurrent seizures. Patients with recurrent seizures were less severely ill according to the APACHE II and SOFA scores, had epilepsy more and metabolic derangements with acidosis less frequently, and were seen more often by consulting neurologists. Presumed underlying etiologies prone to seizures but usually confined to a single organ, such as encephalitis, tended to be more frequent in patients with repetitive seizures (18 patients with repetitive seizures [12.8] versus 3 with single seizures [5.1%]). In contrast, etiologies typically affecting multiple organs but that are usually linked to fewer seizures, such as septic encephalopathy was seen more often in patients with single seizures (9 patients with single seizures [10%] versus 10 with repetitive seizures [7.1%]). Reported seizure semiologies did not differ among patients with and without recurrent seizures except for bilateral seizures that were reported less frequently with recurrent seizures. Patients with recurrent seizures were put on cEEG more frequently and had focal slowing and seizures more often as compared to patients with single seizures (Table 2). To test the hypothesis that a lack of EEG performance may have influenced our secondary outcomes of patients with and without EEG were performed. Spot or continuous EEG 24 h around seizures, however, did not improve outcome or reduce mortality. With spot EEGs 21.1% died versus 20.8% without (*p* = 0.974), and return to premorbid neurologic function at discharge was seen in 23.7% with spot EEGs versus 22.9% without (*p* = 0.913). Similarly, with cEEGs 18.2% died as compared to 22.1% without (*p* = 0.547), and return to premorbid neurologic function at discharge was seen with cEEG in 23.6% versus 23.5% without (*p* = 0.978).

**Table 2:** Univariable comparisons of baseline characteristics of ICU patients with single and recurrent seizures (*n* = 200)

Demographics and admission characteristics	Patients with single seizures (*n* = 59)		Patients with recurrent seizures (*n* = 141)		*p* value*
Age (years; median, IQR)	65	51–75	66	53–75	0.721
Female (*n*, %)	27	45.8	61	43.3	0.745
Charlson Comorbidity Index (median, IQR)	5	2–7	5	3–7	0.717
APACHE II (median, IQR)	26	21–31	23	17–27	**0.003**
SOFA (median, IQR)	7	6–10	6	4–9	**0.010**
Presumed seizure etiology (*n*, %)					
Confirmed potential fatal etiology	49	83.1	112	79.4	0.556
Known epilepsy	7	11.9	36	25.5	**0.032**
Seizure type and characteristics					
Seizures reported to be focal (*n*, %)	14	23.7	62	36.9	0.099
Seizures reported to be focal to bilateral (*n*, %)	38	64.4	60	43.5	**0.005**
Seizures reported with motor symptoms (*n*, %)	41	69.5	82	58.2	0.153
Seizures reported without motor symptoms (*n*, %)	12	20.3	27	19.1	
Seizure reported with altered consciousness (*n*, %)	51	86.4	109	77.3	0.176
Lowest GCS on day of seizure (points; median, IQR)	5	3–9	5	3–11	0.619
EEG 24 h around seizure	30	50.9	122	86.5	** < 0.001**
EEG immediately after or during seizure	21	35.6	103	73.1	** < 0.001**
Continuous EEG	5	8.5	50	35.5	** < 0.001**
EEG with generalized slowing	27	45.8	84	59.6	0.073
EEG with focal slowing	8	13.6	51	36.1	**0.001**
EEG with epileptiform discharges (spikes and sharp waves)	9	15.3	37	26.4	0.101
EEG with epileptic seizures	0	0.0	55	39.0	** < 0.001**
Complications in close temporal relation to seizure (*n*, %)					
Persistent coma for ≥ 24 h following seizure	44	74.6	94	66.7	0.270
Infection at seizure onset	21	35.6	58	41.1	0.465
Acidosis at seizure onset	24	40.7	26	18.4	**0.001**
Seizure following emergency operations	25	42.4	37	26.2	**0.024**
Arterial hypotension requiring vasopressors during seizures	21	35.6	41	29.1	0.364
Treatment characteristics during seizure					
Seen by neurologist (*n*, %)	37	62.7	128	90.8	** < 0.001**
In-hospital treatment (days; median, IQR)	15	8–23	15	9–26	0.996
ICU treatment (days; median, IQR)	4	2–9	6	2–11	0.138
Patients with previous antiseizure drugs (*n*, %)	16	27.1	87	61.7	** < 0.001**
New or additional antiseizure drugs given in response to seizure (*n*, %)	47	79.7	97	68.9	**0.119**
Patients with intravenous anesthetic drugs during seizure (*n*, %)	22	37.3	63	44.7	0.335
Duration of mechanical ventilation (days; median, IQR)	2	1–7	5	2–9	**0.031**
Outcomes (*n*, %)					
No return to premorbid neurologic function at discharge (incl. death)	43	72.9	110	78.0	0.435
Death at hospital discharge	13	22.0	29	20.6	0.816

*IQR* interquartile range, *GCS* Glasgow coma score, *EEG* electroencephalography, *APACHE* *II* acute physiology and chronic health evaluation II (Range 0–71), *SOFA* sequential organ failure assessment (Range 0–24), *ICU* intensive care unit

Bold font indicates statistical significance

*Chi-squared or Fisher’s exact test (for small sample sizes) were used for univariable comparisons of proportions

Among clinical parameters, acidosis at seizure onset or coming out of an emergency operation was seen less often with recurrent seizures (Table 2). While recurrent seizures were not associated secondary outcomes, they were significantly associated with longer mechanical ventilation (median 5 days with recurrent seizures versus 2 days without; *p* = 0.031).

### Multivariable models

Uni- and multivariable analyses including clinical data that were readily available at seizure onset to identify predictors and preventive characteristics for recurrent seizures at the beginning of a seizure are shown in Table 3. Multivariable analyses revealed that acidosis at onset and prior surgery were associated with decreased odds of seizures recurrence independent of all other clinical factors. Of the 50 patients with acidosis at seizure onset, 12 (24% of acidotic patients) had respiratory acidosis without metabolic compensation, and 38 (76% of acidotic patients) had metabolic acidoses not compensated by respiratory efforts. There was no difference regarding the administration of anesthetics between acidotic patients with and without recurrent seizures (11/26 versus 12/24 patients) that would have explained the association between acidosis and decreased seizure recurrence.

**Table 3:** Uni- and multivariable comparisons between seizing ICU patients with and without seizure recurrence

Associations	Univariable				Multivariable		
Potential associations in all ICU patients (i.e., variables being significant in Table 2)	OR for recurrent seizures	95% CI	*p* value*		OR for recurrent seizures	95% CI	*p* value**
Seizures reported to be focal to bilateral	0.41	0.22–0.77	**0.005**		0.60	0.30–1.21	0.154
Epilepsy as presumed etiology	2.55	1.06–6.11	**0.036**		2.25	0.83–6.06	0.110
Metabolic derangement as presumed etiology (other than acidosis)	0.44	0.22–0.85	**0.015**		0.52	0.24–1.09	0.084
APACHE II (per increasing unit)	0.93	0.89–0.97	**0.002**		0.97	0.92–1.04	0.396
SOFA (per increasing unit)	0.88	0.80–0.97	**0.009**		0.98	0.86–1.11	0.707
Acidosis at seizure onset (per decreasing pH)	0.33	0.17–0.64	**0.001**		0.43	0.20–0.94	**0.035**
Previous emergency operation	0.48	0.26–0.92	**0.026**		0.48	0.24–0.97	**0.041**

*OR* odds ratio, *CI* confidence interval, *APACHE II* acute physiology and chronic health evaluation II, *SOFA* sequential organ failure assessment,* ICU* intensive care unit

Bold font indicates statistical significance

*Logistic regression analyses

**Hosmer–Lemeshow goodness-of-fit test: Chi-squared 3.26, *p* = 0.917 indicating adequate model fit

The Hosmer–Lemeshow *χ*^2^ goodness-of-fit tests indicated adequate model fit for our multivariable model.

### Linear regression analysis

**Fig. 2:**
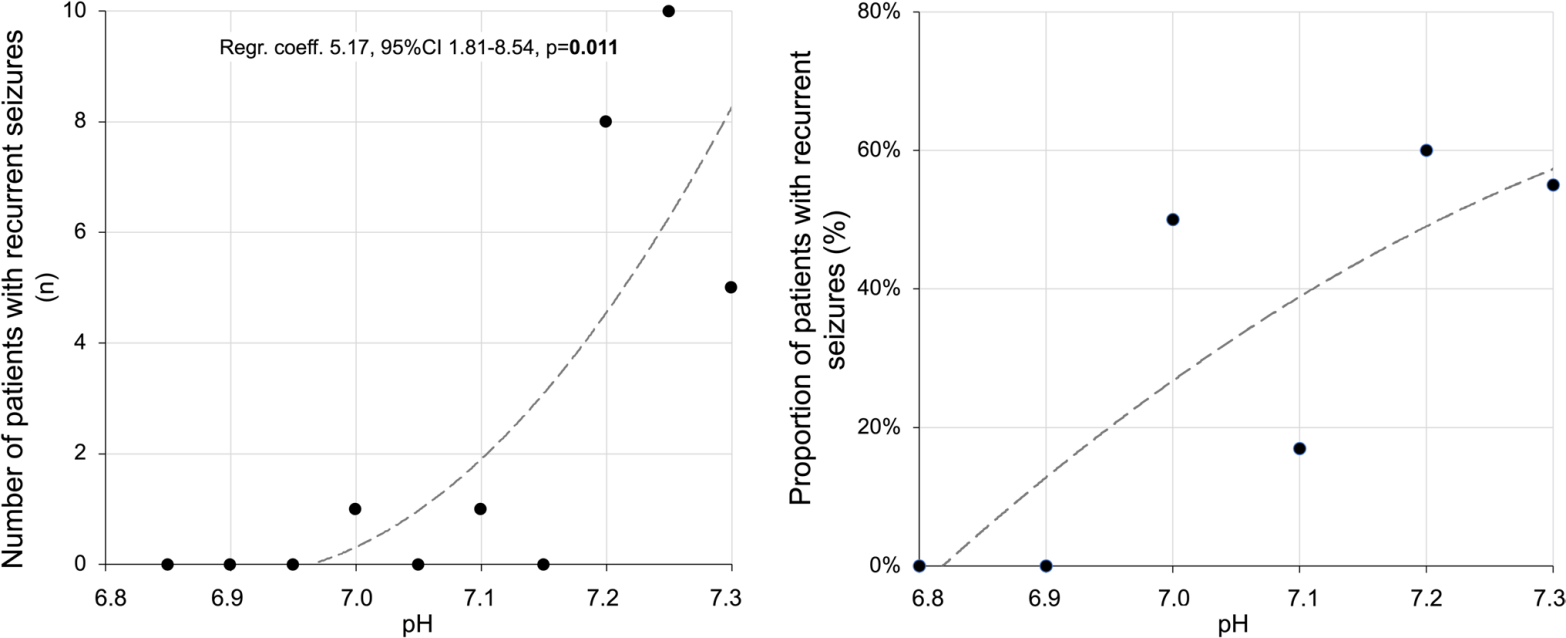
Correlation between the severity of acidosis at seizure onset and occurrence of recurrent seizures

Linear regression analyses revealed a significant correlation between decreasing acidosis at seizure onset and the occurrence of recurrent seizures as shown in Fig. 2.

## Discussion

Our study presents a dataset of a 6-year observation of ICU patients treated in a tertiary academic medical center with neurocritical specialists and readily available EEG. In addition to numerous studies on the clinical impact of status epilepticus, our study reveals that seizures not transforming into status epilepticus can have relevant clinical impact and must be taken seriously. Epileptic seizures in our patients emerged infrequently but in the majority of cases repetitively, and were associated with longer mechanical ventilation, high rates of consultations by neurologists (91%), and an increased use of EEG as compared to patients with single seizures. Although the recurrence of seizures was not associated with secondary outcomes, the increased use of resources (EEG and neurologic consultations, prolonged mechanical ventilation) indicates that early and reliable differentiation between patients with single or recurrent seizures may have an impact not only on optimal individual treatment but also on an economic level. Despite these insights and the strong assumption of an epileptic event by the witnessing clinicians, sound epileptologic workup including EEG was not performed immediately after seizure in every fourth patient. This calls for heightened awareness and a more rigorous workup in such patients.

The low frequency of seizures and the even lower numbers of seizures in our general ICU population contrasts earlier studies reporting higher rates of seizures in general ICU populations ranging from 2.4% [23] in respiratory ICUs, to 3% in cardiac surgery ICUs [4], and 10% in medical ICUs [13]. Our study design cannot exclude an underestimation of the frequency of nonconvulsive epileptic seizures that would have been uncovered with longer and more frequently performed cEEGs. However, this shortcoming is limited as during the study, all patients with clinically unexplained impaired consciousness were rigorously examined by EEG.

The seizure recurrences were frequent (75%), the proportion of patients with seizures in line with more recent studies [12], and the number of cases large enough that our data appear sufficient for determining risk factors for seizure recurrence. Our multivariable models revealed that surgery prior to seizures and acidosis measured in the blood at seizure onset were associated with decreased odds of reoccurring seizures during intensive care. Remarkably, there was no difference regarding the use of anesthetics between acidotic patients with and without recurrent seizures that would have acted as mediators for the association between acidosis and less seizure recurrence. These findings offer interesting discussions of potential pathophysiologic mechanisms. Surgery may be a surrogate for a prolonged postoperative antiseizure effect of intraoperatively administered anesthetics. In contrast, understanding the mechanisms linked with acidosis is challenging. Our analysis revealed an association between acidosis and lower number of patients with recurrent seizures. These findings were mirrored by the correlation between increasing pH and increasing number of patients with recurrent seizures, and animal models describing activation of inhibitory interneurons from acidosis through the acid-sensing ion channel—1a [27]. Further support comes from studies on antiseizure effects of the ketogenic diet, where metabolic acidosis is thought to be an important mediator [10]. In reverse, seizure promoting effects of hyperventilation is likely to trigger epileptic seizures via respiratory alcalosis. Studies on artificially induced mild respiratory acidosis via hypoventilation of mechanically ventilated patients in status epilepticus are lacking but could be worth performing. Besides the accumulation of protons, studies suggest alternative mediators that may accompany acidosis during seizures, such as depletion of oxygen, glutamate, and adenosine triphosphate that can interrupt or suppress seizures [8, 26]. These studies are supported by investigations that suggest reduced levels of these mediators with worsening seizures [5, 7]. Another study on antiseizure effects of acidosis described a reduction of intracellular pH and epileptiform activity of hippocampal CA3 neurons by the carbonic anhydrase inhibitor sulthiame [11]. Other antiseizure drugs with carbonic anhydrase inhibitory properties are topiramate and zonisamide, as well as acetazolamide, the most potent of carbonic anhydrase inhibitors [14]. To what extent blood acidosis of our patients suppressed seizures or represents a surrogate for such alternative pathomechanisms cannot be answered with our study but deserves to be investigated. However, as patients with recurrent seizures were less severely ill according to the APACHE II and SOFA score and had a similar burden of comorbidities, it seems unlikely that acidosis is a surrogate for simply being more critically ill. The association between a low APACHE II and SOFA score and recurrent seizures is likely explained by the distribution of the presumed underlying etiologies of seizures in our cohort. Etiologies that are prone to seizures but usually confined to a single organ, such as for example encephalitis, tended to be more frequent in patients with repetitive seizures. In contrast, etiologies typically affecting multiple organs that may thereby increase the illness severity scores but are usually linked to fewer seizures, such as septic encephalopathies, were seen more frequently in patients with single seizures. However, these results are hampered by small sample sizes and further studies are needed in this context.

Another less surprising finding of our study is the association between known epilepsy and increased odds for recurrent seizures. This association seems plausible, as patients with epilepsy are prone to seizures especially when antiseizure treatment is suboptimal in the context of critical illness or with drugs that may interact with antiseizure medication or their metabolism, as seen with antibiotics [20].

The strength of our study is the large cohort, the observation period of 6 years on a tertiary academic medical care center with a high awareness of seizures in ICU patients, and the use of prospectively monitored and stored clinical data during the entire study period with the digital ICU information system MetaVision (iMDsoft, Wakefield, MA). Another strength lies in the fact that all our ICU patients with clinically unexplained impaired consciousness and/or altered neurologic function were rigorously examined by EEG. Therefore, EEG was lacking only in patients in whom the neurologists and intensivists were reporting motor symptoms typically seen with epileptic seizures.

The single-center observational study design limits the generalizability of our results. However, clinical data regarding treatment, monitoring measures, and complications were collected systematically as routine clinical practice in the ICUs. Due to the retrospective nature of the study, certain nonconvulsive seizures may have been missed and the actual proportion of patients with single and/or recurrent seizures may be higher than reported. A similar limitation comes from the retrospective assessment of reported seizure semiology which was not reported in 11% of patients. In such patients, the EEG had to at least detect epileptiform discharges or epileptic seizures to categorize the patients as having had epileptic seizures. The decision by the treating physicians and neurologists regarding the type of EEG based on the extent of postictal neurologic recovery represents another limitation. Finally, exact data regarding the duration of EEG in cases of cEEG were lacking in a large proportion of patients, so further information regarding the extent of EEG could not be analyzed.

## Conclusions

Seizures in ICU patients are rare, mostly recurrent, and associated with prolonged mechanical ventilation. Whenever seizures are observed in critically ill patients, clinicians should be more vigilant about the high-risk of seizure recurrence and the potential need for prophylactic treatment with antiseizure medications must be carefully discussed on the basis of sound epileptologic workup. While, not surprisingly, known epilepsy seems to promote recurrent seizures, the associations between both acidosis and previous surgery (the latter likely a surrogate for antiseizure effects of anesthesia) with decreased odds for recurrent seizures deserve to be studied more closely. Our results open up the discussion of whether artificial mild acidosis, induced by controlled mild hypoventilation in ventilated patients, has the potential to become another therapeutic option in status epilepticus.

## Data Availability

Anonymized data are available from the corresponding author and will be shared on reasonable request from any qualified investigator.

## References

[1] Baumann SM, Semmlack S, De Marchis GM, Rüegg SH, Marsch S, Sutter R (2020). Frequency and implications of complications in the ICU after status epilepticus. No calm after the storm. Crit Care Med.

[2] Charlson ME, Pompei P, Ales KL, MacKenzie CR (1987). A new method of classifying prognostic comorbidity in longitudinal studies: development and validation. J Chronic Dis.

[3] Garner JS, Jarvis WR, Emori TG, Horan TC, Hughes JM (1988). CDC definitions for nosocomial infections, 1988. Am J Infect Control.

[4] Gofton TE, Chu MW, Norton L, Fox SA, Chase L, Murkin JM, Young GB (2014). A prospective observational study of seizures after cardiac surgery using continuous EEG monitoring. Neurocrit Care.

[5] Haglund MM, Schwartzkroin PA (1990). Role of Na-K pump potassium regulation and IPSPs in seizures and spreading depression in immature rabbit hippocampal slices. J Neurophysiol.

[6] Hirsch LJ, Fong MWK, Leitinger M, LaRoche SM, Beniczky S, Abend NS, Lee JW, Wusthoff CJ, Hahn CD, Westover MB, Gerard EE, Herman ST, Haider HA, Osman G, Rodriguez-Ruiz A, Maciel CB, Gilmore EJ, Fernandez A, Rosenthal ES, Claassen J, Husain AM, Yoo JY, So EL, Kaplan PW, Nuwer MR, van Putten M, Sutter R, Drislane FW, Trinka E, Gaspard N (2021). American clinical neurophysiology society’s standardized critical care EEG terminology: 2021 version. J Clin Neurophysiol.

[7] Kirchner A, Veliskova J, Velisek L (2006). Differential effects of low glucose concentrations on seizures and epileptiform activity in vivo and in vitro. Eur J Neurosci.

[8] Kloiber O, Bockhorst K, Hoehn-Berlage M, Hossmann KA (1993). Effect of hypoxia on bicuculline seizures of rat: NMR spectroscopy and bioluminescence imaging. NMR Biomed.

[9] Knaus WA, Draper EA, Wagner DP, Zimmerman JE (1985). APACHE II: a severity of disease classification system. Crit Care Med.

[10] Kossoff EH, Wang HS (2013). Dietary therapies for epilepsy. Biomed J.

[11] Leniger T, Wiemann M, Bingmann D, Widman G, Hufnagel A, Bonnet U (2002). Carbonic anhydrase inhibitor sulthiame reduces intracellular pH and epileptiform activity of hippocampal CA3 neurons. Epilepsia.

[12] Marcuse LV, Bronster DJ, Fields M, Polanco A, Yu T, Chikwe J (2014). Evaluating the obtunded patient after cardiac surgery: the role of continuous electroencephalography. J Crit Care.

[13] Oddo M, Carrera E, Claassen J, Mayer SA, Hirsch LJ (2009). Continuous electroencephalography in the medical intensive care unit. Crit Care Med.

[14] Ozsoy HZ (2021). Anticonvulsant effects of carbonic anhydrase inhibitors: the enigmatic link between carbonic anhydrases and electrical activity of the brain. Neurochem Res.

[15] Rossetti AO, Hurwitz S, Logroscino G, Bromfield EB (2006). Prognosis of status epilepticus: role of aetiology, age, and consciousness impairment at presentation. J Neurol Neurosurg Psychiatry.

[16] Semmlack S, Tschudin-Sutter S, Widmer AF, Valenca M, Ruegg S, Marsch S, Sutter R (2016). Independent impact of infections on the course and outcome of status epilepticus: a 10-year cohort study. J Neurol.

[17] Silveira DC, Sagi A, Romero R (2019). Are seizures predictors of mortality in critically ill patients in the intensive care unit (ICU)?. Seizure.

[18] Sutter R (2016). Are we prepared to detect subtle and nonconvulsive status epilepticus in critically ill patients?. J Clin Neurophysiol.

[19] Sutter R, Dittrich T, Semmlack S, Ruegg S, Marsch S, Kaplan PW (2018). Acute systemic complications of convulsive status epilepticus-a systematic review. Crit Care Med.

[20] Sutter R, Ruegg S, Tschudin-Sutter S (2015). Seizures as adverse events of antibiotic drugs: a systematic review. Neurology.

[21] Sutter R, Semmlack S, Kaplan PW (2016). Nonconvulsive status epilepticus in adults—insights into the invisible. Nat Rev Neurol.

[22] Sutter R, Tschudin-Sutter S, Grize L, Fuhr P, Bonten MJ, Widmer AF, Marsch S, Ruegg S (2012). Associations between infections and clinical outcome parameters in status epilepticus: a retrospective 5-year cohort study. Epilepsia.

[23] Talwar D, Nair V, Chudiwal J (2012). Seizures in the respiratory ICU: single-center study of patients with new-onset seizures. Crit Care.

[24] Vincent JL, Moreno R, Takala J, Willatts S, De Mendonca A, Bruining H, Reinhart CK, Suter PM, Thijs LG (1996). The SOFA (sepsis-related organ failure assessment) score to describe organ dysfunction/failure. On behalf of the working group on sepsis-related problems of the European society of intensive care medicine. Intensive Care Med.

[25] von Elm E, Altman DG, Egger M, Pocock SJ, Gotzsche PC, Vandenbroucke JP, Initiative S (2007). The strengthening the reporting of observational studies in epidemiology (STROBE) statement: guidelines for reporting observational studies. Lancet.

[26] Yamada K, Ji JJ, Yuan H, Miki T, Sato S, Horimoto N, Shimizu T, Seino S, Inagaki N (2001). Protective role of ATP-sensitive potassium channels in hypoxia-induced generalized seizure. Science.

[27] Ziemann AE, Schnizler MK, Albert GW, Severson MA, Howard MA, Welsh MJ, Wemmie JA (2008). Seizure termination by acidosis depends on ASIC1a. Nat Neurosci.

